# Simple changes to the reporting environment produce a large reduction in the frequency of interruptions to the reporting radiologist: an observational study

**DOI:** 10.1177/02841851221139624

**Published:** 2022-11-27

**Authors:** Carina Banziger, Kirsty McNeil, Hui Lu Goh, Samantha Choi, Ian A Zealley

**Affiliations:** 1 School of Medicine, 7486 University of St Andrews, St Andrews, Scotland, UK; 2 Department of Radiology, NHS Tayside, Ninewells Hospital, Dundee, Scotland, UK; 3 3529 Department of Radiology, NHS Greater Glasgow and Clyde, Glasgow, Scotland, UK; 4 Department of Radiology, 59841 Royal Hospital for Children and Young People, Edinburgh, Scotland, UK

**Keywords:** Computed tomography, interruptions, reporting, reducing interruptions

## Abstract

**Background:**

Interruptions are a cause of discrepancy, errors, and potential safety incidents in radiology. The sources of radiological error are multifactorial and strategies to reduce error should include measures to reduce interruptions.

**Purpose:**

To evaluate the effect of simple changes in the reporting environment on the frequency of interruptions to the reporting radiologist of a hospital radiology department.

**Material and Methods:**

A prospective observational study was carried out. The number and type of potentially disruptive events (PDEs) to the radiologist reporting inpatient computed tomography (CT) scans were recorded during 20 separate 1-h observation periods during both pre- and post-intervention phases. The interventions were (i) relocation of the radiologist to a private, quiet room, and (ii) initial vetting of clinician enquiries via a separate duty radiologist

**Results:**

After the intervention there was an 82% reduction in the number of frank interruptions (PDEs that require the radiologist to abandon the reporting task) from a median 6 events per hour to 1 (95% confidence interval [CI] = 4–6; *P* < 0.00001). The overall number of PDEs was reduced by 56% from a median 11 events per hour to 5 (95% CI = 4.5–11: *P* < 0.00001).

**Conclusion:**

Relocation of inpatient CT reporting to a private, quiet room, coupled with vetting of clinician enquiries via the duty radiologist, resulted in a large reduction in the frequency of interruptions, a frequently cited avoidable source of radiological error.

## Introduction

Contemporary healthcare culture places great emphasis on patient safety. The analysis of error and potential contributory factors is a major focus of healthcare research (1–4). Interruptions are linked to discrepancies, errors, and potential safety incidents in radiology (5,6) as well as in other specialties (7–10). Interruptions increase the time to report for cross-sectional imaging (11), decrease the quality of the work, and increase the perceived workload pressure (12,13). Sources of radiological error are multifactorial: as well as participating in lifelong learning and addressing cognitive bias, strategies to reduce radiological error must include measures to reduce interruptions and other potentially distracting events (14).

The Royal College of Radiologists (RCR) reports a general radiology error rate in the range of 3%–5% but indicates that for some specialized examinations the rate may exceed 30% (15). Radiologists work in an environment of ever-expanding volumes of work, increasingly rapid access to examinations, and quicker turnaround times. In addition to producing imaging reports, radiologists perform a number of additional “non-interpretive” responsibilities that compete for their attention and may interrupt the process of reporting (16).

In the main radiology department at **Ninewells** Hospital, **Dundee**, the principal radiologist reporting inpatient computed tomography (CT) is highly pressured with high volumes of scans and frequent interruptions. With the aim of reducing interruptions, the initial vetting of all clinician enquiries was redirected to the departmental duty radiologist, and the reporting of inpatient CT was relocated to quiet individual reporting rooms.

Recognizing the important impact of interruptions on radiological error, we took the opportunity to perform a pragmatic, opportunistic natural experiment. The hypothesis being tested was that these modifications to the inpatient CT reporting environment would reduce the frequency of interruptions to the reporting radiologist. The aim of the present study was to evaluate the effect of these simple changes on the frequency of interruptions and other potentially distracting events (PDEs) experienced by the inpatient CT radiologist

## Material and Methods

The study was performed after approval from the local Caldicott guardian. No patient identifiable data were recorded.

The pre- and post-intervention elements of the study were performed as prospective observational studies conducted in the radiology department of **Ninewells** Hospital, **Dundee**. The interventions were as follows: (i) initial vetting of all clinician enquiries via the departmental duty radiologist; and (ii) relocation of inpatient CT reporting to private, quiet reporting rooms remote from the busy inpatient CT control area. These interventions took place simultaneously as part of a departmental reorganization and presented the possibility for an opportunistic study into the effect of diverting PDEs away from the reporting radiologist.

During both study periods, 20 separate 1-h observations were conducted. To eliminate sources of bias, inclusion and exclusion criteria were developed (Table 1). All eligible radiologists were of attending grade, and the radiology reporting task being undertaken was “hot reporting” of general medical and surgical inpatient CT scans in a large general hospital. In total, 14 different attending radiologists were observed. Care was taken to distribute the timing of the observations across the five days of the working week and across “office hours” of the working day (Monday to Friday, 09:00 to 17:00) in order to eliminate variations attributable to the beginning or end of shift periods or different days of the week. The first series of 20 separate 1-h observations was performed “pre-intervention” and the second series of 20 separate 1-h observations “post-intervention.”

**Table 1.** Inclusion and exclusion criteria for observations.

	Inclusion criteria	Exclusion criteria
Participants being observed	- Attending grade radiologists	- Radiology registrars, other reporting allied healthcare professionals
Timing	- Monday–Friday between 09:00 and 17:00	- Out of hours
Activity being observed	- Inpatient CT “hot reporting”	- Outpatient CT “cold reporting” - Other imaging modalities

CT, computed tomography.

The observers were two junior doctors intending to pursue a career in radiology and a junior radiology trainee. All of the observers worked to a standardized system, sitting in an agreed location, maintaining complete silence, and using an agreed template for recording PDEs. All the observers attended briefing sessions to ensure understanding of, and compliance with, the observation methodology. The observer was positioned out of the radiologist's line of sight to minimize disruption. During the pre-intervention period, all observations took place in the inpatient CT reporting room, which measured 5 × 3 m. The observer sat in the corner diagonally opposite the reporting radiologist. During the post-intervention period, the observations took place in individual radiologist offices ranging in size from 3 × 3 m to 4 × 3 m. In each office, the radiologist was able to control factors, such as temperature and ambient lighting, to suit their own preference and were in telephone contact with the CT control areas and duty radiologist. Again, the observer sat in the corner diagonally opposite the reporting radiologist.

All PDEs were recorded and categorized following templates adapted from prior studies of interruptions in the anesthetic room environment reported by Salvoldelli et al. and Campbell et al. (Table 2) (7,8). Notes were recorded contemporaneously on structured observation record sheets documenting the time of any PDE, a brief description, the origin, and source. PDEs included anything that may conceivably have distracted or interrupted the radiologist. These were categorized as “potential distractions” (any stimulus that did not result in an observable response or cessation of the active task), “distractions” (resulting in an observable response), or “interruptions” (resulting in cessation of the active task). At the end of each 1-h period of observation, the observer reviewed the record sheets with the observed radiologist to clarify the categorization of individual PDEs.

**Table 2.** Definitions of disruptive events (adapted from Salvodelli et al. (4) and Campbell et al. (3)).

Variables	Definitions
Description	*Potential distraction*: a stimulus that may prevent someone from concentrating *Distraction*: a stimulus that results in an observable response but not cessation of activity *Interruption:* a stimulus that results in period where the radiologist abandons temporarily the vetting, scan supervision, or reporting tasks
Origin	*Internal:* a distracting event that is directly related to the running of the CT service (vetting, scan supervision, reporting) *External:* a distracting event not directly related to the running of the CT service
Source	If *internal*: Team member: any member of the radiology staff also engaged in the running of the CT service Patient: any patient-related factor that distracts the radiologist from vetting, scan supervision, or reporting Workspace environment: any event related to the ergonomics of the CT reporting room Equipment: any failure of the CT scanner, IT infrastructure, or computer systems Insufficient information in requests for CT scans leading to delays in vetting of requests Radiologist-initiated: teaching other members of the staff or students, conversing with other members of staff also engaged in the running of the CT service, conversing with referring clinicians. If *external*: g. External staff: any member of staff who is not directly engaged in the running of the CT service (whether from within or outwith the Department of Radiology) h. Noise: any non-essential conversations or other background noise i. Phones, bleeps, Tannoy announcements j. Other: any other distracting event not included in other classifications

CT, computed tomography; IT, information technology.

## Statistical analysis

The primary endpoint of the study was the observed change in the frequency of “interruptions,” which resulted in the radiologist having to cease their reporting task and then resume it later. As the data were skewed, consolidated data outcomes were reported as median rather than mean values. Using the statistical software SPSS Statistics (IBM Corp., Armonk, NY, USA), the Wilcoxon signed rank test was used to compare total PDEs and interruptions pre- and post-intervention. *P* < 0.05 was considered statistically significant.

## Results

**Fig. 1.**
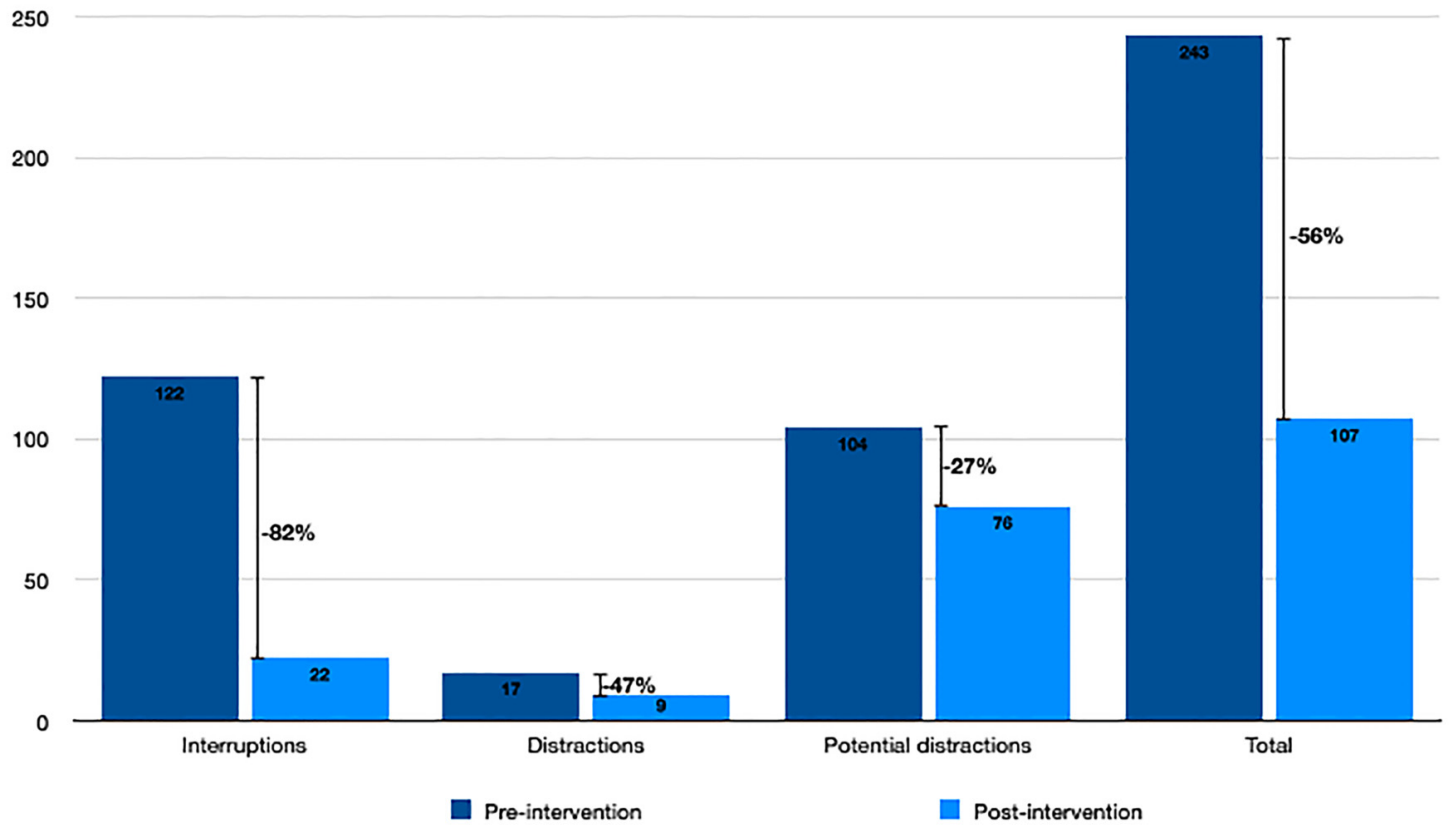
Percentage decrease of potentially disruptive events before and after the intervention.

The number and classification of PDEs recorded in the pre- and post-intervention series of 20 separate 1-h observation periods is tabulated (Tables 3 and 4) and detailed in Fig. 1. The median number of PDEs per hour fell by 56% from 11 to 5 following the introduction of a “duty” radiologist and change of reporting location (*z* = 4.82, 95% confidence interval [CI] = 4.5–11; *P* < 0.00001). Of particular note, the median number of “interruptions” (resulting in cessation of the active task) per hour fell by 82% from 6 to 1 (*z* = 8.20, 95% CI = 4–6; *P* < 0.00001). There were similar reductions in the numbers of distractions and potential distractions.

**Table 3.** Number of PDEs pre- and post-intervention caused by internal sources.

	Source of PDEs											
	Team member		Patient		Workspace		Equipment		Insufficient information		Radiologist initiated	
Observation	Pre	Post	Pre	Post	Pre	Post	Pre	Post	Pre	Post	Pre	Post
1	2	0	2	0	0	0	0	0	0	0	0	0
2	2	2	0	0	0	0	2	0	0	0	0	0
3	2	2	0	0	1	0	3	0	0	0	4	0
4	3	1	0	0	0	0	1	0	1	0	0	0
5	0	1	0	0	0	0	2	0	0	0	1	0
6	1	3	1	0	0	0	0	0	0	0	1	1
7	1	1	0	0	0	0	0	0	0	0	0	0
8	1	0	0	0	0	0	0	0	0	0	2	0
9	2	0	0	0	0	0	0	0	0	0	0	0
10	1	0	0	0	0	0	0	0	0	0	2	0
11	0	2	0	0	0	0	0	0	0	0	1	1
12	0	1	0	0	0	0	0	0	0	0	0	0
13	0	0	0	0	0	0	0	0	0	0	0	0
14	2	1	0	1	0	0	0	0	0	0	0	0
15	2	0	0	0	0	0	1	0	2	0	0	0
16	0	0	0	0	0	0	1	0	0	0	0	0
17	2	1	0	0	0	0	0	0	0	0	2	0
18	2	0	0	0	0	0	1	0	0	0	2	0
19	1	0	0	0	0	0	0	0	0	0	5	0
20	2	0	0	0	0	0	0	0	0	0	0	0
Total	26	15	3	1	1	0	11	0	3	0	20	2
Change (%)	−42		−66		−100		−100		−100		−90	

PDE, potentially disruptive event.

**Table 4.** Number of PDEs pre- and post-intervention caused by external sources.

	Source of PDEs							
	Staff		Noise		Phone/Bleep/Tannoy		Other	
Observation	Pre	Post	Pre	Post	Pre	Post	Pre	Post
1	5	1	3	1	1	5	0	0
2	5	0	10	3	0	1	0	0
3	3	0	5	1	0	7	0	0
4	2	0	4	3	0	8	0	0
5	1	0	0	1	0	0	0	0
6	7	1	0	0	0	0	8	0
7	4	0	5	1	0	0	0	0
8	0	1	3	0	0	2	0	0
9	2	1	3	1	1	3	0	0
10	2	2	1	0	5	3	0	0
11	2	1	0	0	1	6	0	0
12	5	1	15	1	0	0	0	0
13	0	0	15	0	1	0	0	0
14	4	0	11	1	2	1	0	0
15	2	0	5	0	1	0	1	0
16	4	0	11	0	0	0	0	0
17	3	0	7	0	2	3	0	0
18	4	3	2	0	0	4	0	0
19	3	0	0	0	0	7	0	0
20	1	0	5	0	1	7	0	0
Total	59	11	104	13	15	65	1	0
Change (%)	−81		−88		+433		−100	

PDE, potentially disruptive event.

During the post-intervention period, there was a decrease in all “internal” PDEs arising from radiology team members, patients, workspace issues, equipment, and inadequate clinical information (Table 3). Notably, during the pre-intervention period when the radiologists were using shared workstations, there were 11 episodes of workstation failure requiring abandonment of reporting task and rebooting, whereas there were no episodes of information technology (IT) equipment failure during the post-intervention period when the radiologists were using dedicated individual workstations.

Post-intervention, there was a decrease in most “external” PDEs including external staff, noise, and “other” (Table 4). However, there was an increase in those PDEs categorized as “phones, bleeps or Tannoy announcements” from 15 events in 20 h pre-intervention to 65 events post-intervention, the vast majority of which were Tannoy announcements (n = 63, 87%).

A post hoc power calculation was performed for the primary endpoint of change in the frequency of “interruptions,” those PDEs resulting in cessation, and resumption of the reporting task. This demonstrated that the study was powered at >95% to detect the observed 82% reduction at a significance level of *P* < 0.01. On this basis, the null hypothesis was rejected, and the hypothesis that the stated modifications to the inpatient CT reporting environment have resulted in a reduced frequency of interruptions to the reporting radiologist was accepted.

## Discussion

The increased frequency of interruptions is associated with increased rates of radiological error (5,6). Reducing interruptions reduces the radiologist’s workload pressure and increases reporting and efficiency, all factors contributing to improved patient safety (17).

Distraction during performance of complex tasks is a recognized source of error in radiology and many other disciplines (5–10,18). Aviation employs “sterile cockpit” rules during critical phases of flight to avoid disruptions. This has led to the application of sterile cockpit rules to the delivery of some complex healthcare delivery where there is a risk of causing harm (19). Inpatient CT scans are often complex and challenging to interpret and report accurately. The inpatient CT area in our department is a distraction-rich environment. Executing complex tasks in this environment is an avoidable source of error.

This study has demonstrated a substantial beneficial effect from simple changes to the reporting environment: the initial vetting of clinician enquiries via the duty radiologist and the relocation of reporting to quiet, private reporting rooms. These changes results in an 82% reduction in the rate of “interruptions,” events that caused the radiologist to completely cease their reporting task and subsequently to resume it later. There were also fewer “potential distractions” and “distractions,” resulting in an overall reduction of PDEs of 56%.

Almost all sources of disruption were reduced in the post-intervention period. An exception was the external source “phone, bleep, and Tannoy,” most of which were attributable to Tannoy announcements (63 events compared with 3 pre-intervention). This increase in the use of the Tannoy system was attributable to other changes in departmental workflow that took place at the same time as the departmental reorganization, which resulted in changes to the reporting radiologist environment, and which precipitated this opportunistic study. The increase will be addressed in the department to further reduce the rate of PDEs for all staff.

There was a 100% reduction in PDEs relating to equipment and IT issues. We hypothesize that the use of shared IT equipment in the pre-intervention period is associated with more frequent software failure and other similar glitches. In the pre-intervention period, there were 11 episodes of equipment failure requiring abandonment of the reporting task and re-booting of workstations. Over the study period, no appreciable changes were made to the workstations, software, and other IT infrastructure. However, the workstations in the inpatient CT reporting area that were used during the pre-intervention period were shared, whereas the equipment used in the post-intervention period were dedicated workstations for specific radiologists in private reporting rooms.

Previous studies of disruption to radiology workflow report telephone calls as a frequent cause of interruption, with one study recording an average of 72 telephone calls, lasting a total of 2 h, during a 12-h on-call shift (6,16). The probability of a telephone call interrupting the reporting of an examination that takes 5 min to report was 37%, increasing to 59% for a 10-min examination: this reported frequency of interruption did not even take into account disruptions arising from interactions with staff members of the radiology department and clinicians. The present study was performed during office hours with telephone calls accounting for 16% of all PDEs, 65% of them being directly related to the running of the CT service.

There is a clear relationship between interruptions and increased risk of error in radiology. Balint et al. (5) reported that an increase of one telephone call over the average per hour was associated with a 12% increase in the likelihood of a radiology resident's provisional report including a “significant error,” defined as a change in final diagnosis requiring direct communication with the clinician (5).

Interruptions not only create potential for error due to forgetting but also introduce time delays as individuals must work to retrieve memories, or repeat work previously done. This is the so-called “resumption lag” that results from switching tasks, and the requirement to re-orient oneself on resumption (3,20,21). Distractions occurring during the assessment for lung nodules on CT chest examinations prolong reporting times (11). It has also been reported that a loss of concentration caused by interruptions during the performance of complex tasks, such as reporting cross-sectional studies, results in a failure to report abnormalities previously identified but forgotten during the period when the radiologist's attention is diverted (22).

The intensity of PDEs has a pronounced effect on cognition. A study investigating cognitive performance in demanding situations reported rates of forgetting over brief periods (up to 40 s) of 8% in standard conditions, 20%–25% in demanding conditions, and 30%–40% when interruptions occurred (23).

It is not possible to quantify accurately the effect that any single PDE has on a radiologist since no specific objective measurement can be made. The radiologist may not be aware of any effect or may be unable to gauge the consequences of any specific PDE. This is especially the case for “potential distractions,” stimuli that did not result in an observable response or cessation of the active task. These minor disruptions to the reporting environment accounted for 43% of PDEs in the pre-intervention period when the frequency of frank interruptions was high, and 71% in the post-intervention period, when there were far fewer frank interruptions. It is not possible to determine whether these PDEs had an internal effect on the radiologist; we can merely observe that no visible response was elicited. Events of this type may have little or no effect on the radiologist and the active task in hand, but equally they may cause difficult-to-quantify adverse effects that are neither observable nor perceived by the radiologist

One important factor that warrants specific mention is the importance of clinical-radiological discussion, particularly when reporting complex examinations. The frequency of interruptions arising from external (non-radiology) staff did fall during the post-intervention period, but it was perceived that this was due to a reduction in interactions related to CT scan scheduling and prioritization rather than scan interpretation. The tasks of scheduling and prioritization were instead being performed by the duty radiologist and the radiographer staff without interrupting the reporting radiologist. The modifications to the inpatient CT reporting environment that we describe did not prevent clinicians from visiting or telephoning the department to discuss specific cases with the reporting radiologist. Indeed, one advantage was that clinical-radiological discussions could take place in a more peaceful setting, with fewer distractions and interruptions arising from the surrounding environment.

The present study has some limitations. These are related to the duration of the observation periods and to the risk of misclassification of PDEs. The pre- and post-intervention periods of observation were limited to 20 separate hours of observation that may have introduced the possibility of bias due to under-sampling. However, a post hoc power calculation derived from the observed data demonstrated that the reported change in the primary endpoint is very likely to be a real effect, with a >95% likelihood of detecting the observed 82% reduction in interruptions at a significance level of *P* < 0.01. It is also possible that there may have been discrepancies between the observer and the radiologist in the classification of PDEs: for example, a conversation with a colleague may not be classified as an interruption if no active reporting task was being undertaken at the time. To address this potential source of bias, we included a brief discussion between the observer and the radiologist at the end of each observation period during which the classification of PDEs that had been recorded during the session were clarified and agreed upon. Finally, the study did not address directly the relationship between the frequency of interruptions and rates of radiological error. The generation of universally accepted rates of radiological error, and their classification, is a problematic area of research in radiology. For example, reported radiological discrepancy rates vary widely at 5%–9% for emergency department plain radiographs and at 31%–37% for oncological CT imaging (22). There is large variation between radiologists when classifying the “severity” of radiological errors (24,25). Further research into the impact of interruptions, and measures designed to reduce interruptions, on rates of radiological error is desirable. The ideal study of this type would include a range of different departments collaborating in a prospective study, working to an agreed methodology for the identification and classification of errors. However, any such study would face the substantial challenge presented by the lack of agreement between radiologists when attempting to classifying radiological errors (24,25).

In conclusion, simple changes to the reporting environment, initial vetting of clinician enquiries via the duty radiologist, and relocation of inpatient CT reporting to quiet, private reporting rooms resulted in an 82% reduction in the frequency of interruptions to the reporting radiologist from six per hour to one per hour. With interruptions as a frequently cited source of radiological error, these simple changes may help to reduce the risk of error associated with radiology reporting.
